# Puerperal Scarlatina

**Published:** 1876-10

**Authors:** 

**Affiliations:** Halle


Puerperal Scarlatina.—*By Professor Olshausen, of Halle.*—If we estimate the facts given by McClintock in reference to the relation between the mortality of the disease and the date of its onset, the difference becomes still more striking. For McClintock informs us that of sixteen patients who were attacked after the second day only two died, and both of these fell ill on the third day.

Out of fifty-six cases death occurred six times on the second or third day after delivery; thirty-three times between the fourth and seventh; six times on the eighth or ninth; nine times between the tenth and fifteenth; and twice still later.

With regard to the course and symptoms of the disease, it is repeatedly recorded that the eruption spreads rapidly and almost instantaneously over the whole body. This was the case also with all my patients. The eruption also almost always followed rapidly upon the commencement of the fever. More rarely a fever of from one to two days' duration preceded the appearance of the rash. Many observers have been struck by an intense redness of the face even before the onset of the eruption. In my two last cases it was very well marked, and lasted several days. The eruption on the trunk in severe cases generally assume a livid, bluish-red color; and in those which end fatally, usually maintains this color throughout if death follows within a week. We may find some analogy between the intensity and deep-red color of the scarlatinal eruption in the puerperal state, and the frequent occurrence of variola hæmorrhagica in puerperal women. Mention also is made by many observers of the occurrence of sudamina. It is to this scarlatina miliaris especially that some have wished to ascribe a septicæmic character, although it is nothing else than ordinary scarlatina with sudamina, which are so readily produced by perspiration in puerperal women. Women who suffer from typhus fever after delivery also often show sudamina, and are distinguished from other typhus patients by their moist skin during the first stages of the disease.

The chief peculiarity noticed by all observers is the slight character of the sore throat, which often causes no distress at all, and in many instances of undoubtedly genuine scarlatina is described as almost entirely absent. Among the symptoms mention should be made of diarrhœa, as being of frequent occurrence and unfavorable import. McClintock gives special warning against the administration of aperients, which have an unusual tendency to set up fatal diarrhœa. I have myself experienced this in one case. There can be no doubt about the ominous significance of this complication, for out of fifty-eight cases, in which there is no mention of diarrhœa, twelve died; while out of twenty-one with diarrhœa, fifteen died.

The usual functions appertaining to the puerperal state, such as lochia, secretion of milk, involution of the uterus, proceed, in the great majority of cases, without any disturbance. Only a moderate tenderness of the uterus, which rapidly disappears, is noticed in a small number of cases quite at the commencement of the scarlatina. Inflammatory affections of the pelvic organs are of the greatest rarity, and are only to be regarded as casual complications. Whether the peritonitis which McClintock observed in two cases to make its appearance about the time of desquamation had any etiological relation with the scarlatina must remain uncertain. No mention is made anywhere of hæmorrhage from the uterus, such as occurs in variola or typhus after delivery. The outbreak of scarlatina during pregnancy led, in at least four cases out of seven, to premature delivery.

With regard to treatment there is little to be said. Purgatives are to be avoided, as has been already stated. Stimulants, as recommended by Halahan, in the severe cases, are certainly advisable. But, above all, we must in the present day not omit to administer lukewarm or cold baths if the fever continues at a high point.

If we ask ourselves once more what valid reasons there were for regarding the puerperal diseases which appeared in the guise of scarlatina as being anything else than genuine scarlatina, we can only answer—none whatever. Neither the manifestations of the disease nor the post-mortem lesions, putting aside some quite exceptional cases, had any resemblance whatever to puerperal septicæmia or pyæmia; while, with slight modifications, which, however, are generally or universally present (slightness of sore throat, frequency of miliary vesicles), the typical picture of scarlatina is completely portrayed. Where, we must further ask, in epidemics of puerperal fever have cases been seen with the usual symptoms and post-mortem lesions of peritonitis, and combined with this an eruption resembling that of scarlatina? No case of the kind is to be found in literature. For what Retzius describes was an epidemic of erysipelas, is represented as such even by himself; and, according to the description, had no resemblance at all to scarlatina. So the disease seen by Kiwisch was something quite different. The "circumscribed, generally bluish-red swellings, often only as large as a kreuzer, on the backs of the hands and feet," may point to embolism, or may have depended upon venous thrombosis. But anything resembling scarlatina it is clear that Kiwisch has never observed, or, at any rate, never described. Nevertheless, he makes a sweeping assertion, that the puerperal scarlatina of other authors was a local manifestation of puerperal fever, and puts the same opinion into the mouth of Malfatti, although Malfatti himself quite explicitly declares that he had to do with genuine scarlatina. Also Hodge is said by Kiwisch to have observed a scarlatina-like puerperal fever—a statement which is repeated by Winckel and others; although Hodge, in the article quoted, says not a single word of any eruption whatever, but describes cases of quite ordinary puerperal septicæmia. Thus, through the false statements and representations of certain individuals (especially through those of Helm and Kiwisch, and on the strength of the latter's high authority), the doctrine has acquired shape, that in puerperal fever scarlatina-like eruptions often occur. All later authors repeat this view without bringing forward any evidence in its favor. And even Schroeder expresses his opinion that the majority of cases described as scarlatina after delivery were really of quite a different nature.

Whoever studies the literature of the subject can, I am convinced, come to no other conclusion than that the whole doctrine is false, that it has been accepted without any ground upon the authority of certain individuals, and that so it has crept from one text-book to another.

The two points which may perhaps have first given rise to the suspicion that the so-called scarlatina was really one of the deadly puerperal diseases, are certainly the great mortality of the cases, and the almost constant commencement of the disease within the first three days after delivery. To these two points we now return. The first fact—namely, the greater danger of scarlatina in puerperal women, as compared with other persons—we are indeed unable to explain, but we find in it no ground for the suspicion which has been expressed. We see, indeed, the same thing with respect to the blood diseases, especially small-pox, when they occur after delivery. The frequency with which the severe, and especially the hæmorrhagic forms of this disease occur in puerperal women has been frequently recorded, and came forcibly under my own notice in the epidemic of small-pox which prevailed here in the year 1871, in which twenty-eight pregnant and puerperal women were treated in the lying-in wards. The same thing seems to hold true of typus. Murchison regards typhus in the advanced stages of pregnancy as almost inevitably fatal. Ziemssen lost six out of eighteen pregnant women with typhus, and three out of seven after delivery. We must therefore recognize the fact of the greater danger of scarlatina after delivery, and, in accordance with the analogies which I have mentioned, we may find it at least intelligible.

The second fact, that the scarlatina in about four-fifths of the cases makes its appearance within the first three days after delivery, is generally explained by those who, like Hervieux, have paid especial attention to this circumstance, as being due to an unusually short incubation of scarlatina in puerperal women. Hervieux relates that many who had entered the hospital while pregnant were attacked on the day of delivery itself, or on the following one. This fact however admits of another explanation, which is a much more probable one—namely, that the infection had already been communicated before delivery, but that the disease did not break out until after the confinement. If now we take into consideration the fact that in most cases no opportunity of infection could be demonstrated within quite the last period of pregnancy, but that the outbreak of the disease took place within the first three days after delivery, we are led to the explanation that during pregnancy the outbreak is extremely difficult, but that after delivery it is unusually easy. That, as a mere coincidence, in such a large number of cases the infection was always communicated just at the end of pregnancy, as Schneider maintains, is altogether improbable. We are therefore driven to the conclusion that, during pregnancy, the incubation period is generally prolonged, and under some circumstances may perhaps last for months, until, as soon as delivery has occurred, the contagium becomes active. In fact, various circumstances combine to support this view. Foremost among these I reckon the fact which cannot be doubted, that during pregnancy scarlatina is very rarely observed. In the whole extent of literature I have been able to find only the seven cases mentioned, while within the first week after delivery we have one hundred and thirty-four cases. Senn, Tourtual, and Trousseau expressly declare that in widespread epidemics of scarlatina they have never seen a pregnant woman attacked. Dance and Hervieux saw only one case amongst many which occurred in puerperal women. McClintock, Halahan, and Braxton Hicks, with their wide experience of scarlatina after delivery, say not a word of scarlatina in pregnant women.

There are, moreover, a considerable number of individual cases which tell in favor of the view which I have expressed. Montgomery (quoted by Tanner, "The Signs and Diseases of Pregnancy," 1867, p. 338) relates that a pregnant woman in the ninth month of pregnancy nursed her brother, who was suffering from severe scarlatina. She herself fell ill on the day after delivery, and died after some days. Unfortunately, the exact dates are not given, at any rate by Tanner. Yet Montgomery himself draws the conclusion from this and similar cases, that the infection does not produce its effects until after delivery, even when it has been communicated long before.

The facts communicated by Braxton Hicks are of more importance. A pregnant woman had nursed her children in scarlatina a month before delivery. She fell ill on the second day after delivery, and died on the fifteenth day. Two others (Cases 8 and 11) had nursed their children in scarlatina, one ten days, the other several weeks previously, and both fell ill within the first few days after delivery. The children of another, who was attacked on the third day after delivery, had two months before suffered from slight fever with a scarlatina-like rash. These cases are well adapted to support the hypothesis of the prolonged incubation; and Hicks is led by his experience to a much greater certainty than Montgomery, that this is the true explanation. He regards it as undoubted, that scarlatina in pregnancy does not, as a rule, come to an outbreak, but remains in the incubative stage for months.

This conclusion, as being one which contradicts the views hitherto held as to incubation, is likely at first to meet with little approval. But the facts support the conclusion, and such experiences as those in my two last cases, in which the possibility of infection by scarlatina poison during the last few weeks of pregnancy was almost excluded, tend to confirm it. Perhaps many a case of scarlatina in patients who are not pregnant, which we now regard as proving the persistence of the scarlatinal poison, will hereafter be naturally explained by a long duration of the incubation period. As to the reason why in the case of pregnant women the disease very rarely comes to an outbreak, while yet the poison persists, and remains latent for a long time, we must abstain from forming any hypothesis, since not only the nature of the contagium but the cause of the incubation in acute infectious diseases is completely unknown to us.

It is, however, not impossible that the same relation which scarlatina has to pregnancy may hold true also, although in less degree, for typhoid fever. Montgomery holds very decidedly the same view with regard to this disease. He relates the following: A pregnant woman, in the eighth month, nursed her husband in typhoid fever. After his recovery, she went to visit her father, at whose house she was delivered five or six weeks later, was attacked by typhoid immediately afterwards, and died at the end of a week. Although a single case proves little, and although it is certain that it is not very rare for typhoid to occur during pregnancy, yet it seems that it is very general for typhus or typhoid, when it attacks lying-in women, to make its appearance within the first few days after delivery. This was so in three cases observed by Sinclair and Johnston, in which the disease began respectively on the first, second, and third day after delivery; also, in a case recorded by Breslau, in a case of Winckel's, in two cases of

Hecker's, and in one case observed by myself, which has not been published. In all these cases the disease made its appearance on the third day at latest. Although such a small number of cases—which may perhaps, if literature be searched, be met by an equal number in which the onset was later—may not prove much, yet published observations are always worthy of attention, and it will be worth while in future to direct special notice to this point, whether a somewhat similar rule to that for scarlatina does not hold good with regard to typhus and typhoid. Although the immunity, with regard to these diseases, during the latter part of pregnancy, which Rokitansky assumes may not really exist, yet women under such circumstances appear to be much less easily attacked than those who are not pregnant, a circumstance which may perhaps be interpreted thus—that the disease not unfrequently remains latent until after delivery.

Although an incubation of scarlatina, prolonged through several months, may at first sight appear improbable, and contrary to all our present views as to the incubation of acute exanthemata, yet an analogy for this may be found in the malaria poisons. For it had been already shown by older observations, and has been positively confirmed by the experience of Braune, Fiedler, and Rheinstadter, that the malaria poison may remain latent for from several months to a year—that is to say, that in a previously healthy person an intermittent fever may, under some circumstances as yet unknown, break out for the first time a year after the infection.

As I have already said, the origin of many an obscure case of scarlatina may perhaps hereafter find a new explanation, if we admit and take into consideration the possibility of a long incubation.

I cannot close this article without making a very brief reference to the important discussion on puerperal fever, which took place last year at the Obstetrical Society of London. A debate on the relation of puerperal fever to other diseases, in which the most prominent members of the Society took part, was prolonged through four long meetings, and most astonishing views were therein brought to light. Several speakers are of the opinion that, from the infection by the poison of scarlatina, typhus, and other diseases, lying-in women may get puerperal fever, and indeed that this is no uncommon occurrence. Newman (of Stamford), and Braxton Hicks, are the most uncompromising advocates of this view. The latter even maintains that a lying-in woman infected with scarlatina poison gets puerperal fever, and that, if persons not pregnant again take the infection from her, the disease appears afresh as scarlatina. Leishman and Playfair express themselves more cautiously, and say that lying-in women, by the infection of scarlatina poison, may acquire such a disease that, at any rate in its later stages, their condition cannot be distinguished from that which is found in puerperal fever. These views met with strong contradiction in the Society. Squire especially expresses a decided opinion that puerperal fever has nothing to do with scarlatina, typhus, or any other acute exanthem. He and Huntley maintain that scarlatina appears in puerperal women under the same form as apart from the puerperal state, and therefore can be readily diagnosed as scarlatina. Savage, Williams, Snow Beck, Farre, and West also give their adhesion to this view. The identity of puerperal fever with septicæmia is only lightly touched upon throughout the whole discussion. Fordyce Barker (of New York) does not believe in the identity or near relationship of these conditions. Brunton does not believe in the origin of puerperal fever through infection from the poison of post-mortem examinations, erysipelas, or the like. He believes only in the autogenetic origin. And this in the year 1875! Did not then Semmelweiss, fully a generation ago, make known his discovery, and support it by incontrovertible proof?

Supposing that Braxton Hicks were right in believing that the poison of scarlatina may produce in lying-in women a disease identical even in its anatomical characters with that which we are accustomed to call puerperal fever, we should even then only be able to infer an etiological relationship between the two diseases, but scarlatina would still remain scarlatina, and puerperal fever puerperal fever. In the same way we do not regard a phlegmonous erysipelas, by the ichor of which a puerperal septicæmia is produced, as identical with the puerperal fever. It is only the material which originates the disease which is the same.

The discussion in the London Society has, moreover, again shown how much more experience of scarlatina after delivery English doctors have than we. The explanation of this is, in my opinion, obvious. Were it the custom with us, as it is in England, that normal confinements should be managed and attended by medical practitioners, more lying-in women amongst us also would be infected with scarlatina. Hicks mentions expressly in his paper, published in the year 1871, that in several of his cases the family doctor who had attended the confinement had at the same time cases of scarlatina under his care, and had probably infected the pregnant woman.

I hope that this article may lead to the result that in future, in the observation of cases of scarlatina after delivery, the question of the incubation may be especially tested, and that so the evidence which I have here brought forward may be extended and completed. I am convinced that the doctrine of a "scarlatina puerperalis," in the sense used by Helm, will then be no more heard of.

*Postscript.*—Since I sent this article to the press, a paper on the same subject, by A. Martin, has appeared in the *Zeitschrift fur Geburtshulfe und Frauenkrankheiten*, Bd. i. Heft 2. A. Martin relates three new cases of genuine scarlatina after delivery. All three ended fatally. In one case only there appeared a peritonitis, which, as in McClintock's case, first manifested itself at a late stage, when desquamation was already commencing. Martin himself regards it as a secondary result of the scarlatina. In the two other cases, which ran their course without any peritonitis, there were found after death endometritis, parametritis, and once also lymphangitis. The author expresses the opinion that the prognosis in puerperal scarlatina is really dependent upon the occurrence of secondary lesions in the genital organs—an opinion which will at once be contradicted by a careful examination of literature. When Martin further says that those scarlatina-like processes, which make their appearance at the height of an ichorrhæmic disease, are to be regarded as erysipelas, we can entirely agree with him. But such processes, which especially occur in puerperal pyæmia, have so little resemblance to scarlatina, except at their very first commencement, that they can scarcely be mistaken for it. This will be seen by an examination of the descriptions of Kiwisch and others, and also of the epidemic of erysipelas among lying-in women described by Retzius.

When an eruption in a lying-in woman has the appearance of that of scarlatina, we may boldly regard it as genuine scarlatina, so long as the accompanying symptoms and the course of the disease are not analogous to a puerperal septicæmia or pyæmia, an analogy which cannot be inferred from the descriptions either of Malfatti or Helm. That erysipelas occurs in lying-in women, either alone or in company with other constitutional diseases, that in rare cases epidemics of erysipelas arise, and that further limited inflammation of the skin occurs, especially in the neighborhood of the joints, cannot be denied.

The question of the incubation is not touched upon by A. Martin.—*Archiv fur Gynakologie—Obstet. Jour.*

